# AMGST: Adaptive multi-graph convolution and spatiotemporal attention network for traffic forecasting

**DOI:** 10.1371/journal.pone.0342235

**Published:** 2026-06-04

**Authors:** Pei Shi, Qixiang Lu, Jiahui Chen, Xiaoliu Lv, Lu Zhang, Liang Kuang, Jiadong Sun

**Affiliations:** 1 School of IoT Engineering, Wuxi University, Wuxi, China; 2 Jiangsu Engineering Research Center of Hyperconvergence application and security of IoT devices, Wuxi, China; 3 School of Electronics & Information Engineering, Nanjing University of Information Science & Technology, Nanjing, China; 4 Jiangsu Vocational College of Information Technology, Wuxi, Hebei, China; National University of Defense Technology, CHINA

## Abstract

Traffic forecasting is crucial for optimizing traffic management and control strategies. As a powerful approach for analyzing and mining graph-structured data, graph convolution has shown great potential in traffic prediction. However, it still struggles to fully capture global spatial correlations and long-term dynamic temporal dependencies inherent in spatiotemporal traffic patterns. Moreover, the quality of the graph structure directly affects the extraction of these correlations. To address these challenges, we propose AMGST, an Adaptive Multi-Graph Convolution and Spatiotemporal Multi-Head Self-Attention Network for traffic forecasting. AMGST integrates an Adaptive Spatiotemporal Embedding (ASTE) generator, a multi-graph diffusion convolution module, and a spatiotemporal attention mechanism. First, dynamic spatiotemporal representations are generated using the ASTE module. Then, the multi-graph diffusion convolution leverages both a maximum mutual information coefficient matrix and an adaptive matrix to extract fine-grained spatial features. A global spatial attention mechanism is applied to capture dynamic spatial correlations, while a temporal attention module models nonlinear temporal dependencies. Experimental results on four public traffic datasets, including both speed and flow measurements, demonstrate that AMGST consistently surpasses the baselines, confirming its effectiveness in providing accurate traffic forecasts.

## 1. Introduction

With the rapid advancement of urbanization, traffic congestion has emerged as a pervasive issue, significantly impacting people’s daily lives and work. Traffic data is a typical spatiotemporal time series. Accurate traffic prediction can significantly enhance the optimal control and scheduling of urban transportation, thereby improving the convenience of daily activities. In traffic prediction research, it is essential to consider both the temporal and spatial aspects of traffic data. Graph Convolutional Networks (GCNs) are widely used in this field due to their powerful capability to extract features from graph-structured data.

Traditional GCN-based prediction methods typically rely on adjacency matrices constructed from physical distances or connectivity to extract spatial information. [1–3] However, these methods are limited in their ability to capture only local spatial dependencies, as the adjacency matrix does not account for relationships between non-adjacent nodes. To address this limitation, some researchers have employed techniques such as Dynamic Time Warping (DTW) and Pearson correlation [4] to build new graph structures capable of capturing long-range spatial dependencies. Additionally, the spatial relationships between nodes are inherently dynamic. External factors such as weather, congestion, and accidents can influence traffic flow and alter spatial traffic patterns, which static adjacency matrices fail to capture. Consequently, researchers have explored dynamic graph convolution methods to extract these dynamic spatial dependencies. Although these approaches have improved prediction accuracy to some extent, they still tend to overlook fine-grained spatial information in traffic data, limiting further enhancements in prediction performance.

Temporal Convolutional Networks (TCNs) are widely used to model the temporal dependencies in traffic data. TCNs expand the receptive field by stacking multiple convolutional layers. However, deep stacking may lead to issues such as over-smoothing, increased computational overhead, and gradient explosion. Wu et al. [5] employed TCN and GCN with dilated causal convolution to extract spatiotemporal dependencies in traffic data, and used Graph WaveNet to reduce the number of layers while maintaining the same receptive field. Nevertheless, TCNs are still constrained by their receptive field and are often ineffective in capturing long-term temporal dependencies effectively.

To overcome these limitations, Guo et al. [6] proposed Attention-based Spatiotemporal Graph Convolutional Networks (ASTGCN), introducing an additive attention mechanism after the spatiotemporal graph convolution blocks to extract long-term spatiotemporal dependencies. Liu et al. [7] proposed a Spatiotemporal Autoencoder (ST_AE), which projects extracted spatiotemporal features into a hidden state and applies a self-attention mechanism after the encoder to achieve strong long-term prediction performance. Zheng et al. [8] proposed the Graph Multi-Attention Network (GMAN), which directly uses spatiotemporal self-attention to capture spatiotemporal features. Yan [9] introduced GECRAN, which incorporates an attention module after the Graph Convolutional Recurrent Network to capture long-period dependencies in traffic sequences, achieving excellent results. However, similar temporal patterns can span multiple time steps. Although these studies have yielded promising prediction results, they often overlook the long-term temporal dependencies between spatially distributed nodes across different time steps.

Additionally, traditional traffic prediction models tend to rely on stacked spatiotemporal modules to extract features, resulting in complex architectures, an excessive number of parameters, and limited predictive performance. GdFormer [10] is an attention-based network built on an encoder-decoder structure that utilizes self-attention and diffusion attention to extract spatiotemporal correlations in traffic flow. T-DGAN [11] adopts a Transformer-based encoder-decoder architecture, incorporating spatiotemporal convolution blocks and diffusion attention mechanisms to extract spatiotemporal dependencies and dynamic spatial correlations, respectively. Although these models yield some performance improvements, they overlook the importance of embedding, which are essential for enhancing the effectiveness of attention mechanisms. As a result, their ability to capture complex spatiotemporal features remains limited. In contrast, models such as GMAN and RGDAN [12] incorporate spatiotemporal embeddings into their frameworks, thereby enhancing their predictive performance. Shao et al. [13] proposed the Spatial-Temporal Identity (STID) model, which achieved excellent results using simple spatiotemporal embeddings combined with a multi-layer perceptron. However, these methods still fall short in capturing long-term spatiotemporal dependencies in traffic data, and their predictive capabilities require further improvement.

To address these challenges, this paper proposes an Adaptive spatiotemporal embedding multi-graph convolution Spatiotemporal attention model, namely AMGST, for traffic data prediction. To capture the key spatiotemporal correlations in traffic data, we introduce an adaptive embedding generator to represent input features. Considering inter-node relationships and integrating fine-grained spatial information, we design a multi-graph diffusion convolutional network to extract spatial features using both static and dynamic adjacency matrices, allowing the model to capture richer spatial information in traffic networks. To capture the long-term spatial and temporal dependencies of traffic data, we incorporate a spatiotemporal multi-head self-attention mechanism, which effectively extracts dynamic spatial dependencies and nonlinear temporal correlations in the data. Our contributions can be summarized as follows:

1. AMGST introduces a multi-graph diffusion convolution module that leverages the Maximum Mutual Information Coefficient matrix and adaptive matrix to capture both local and latent spatial dependencies, allowing the model to mine fine-grained spatial features in traffic data.
2. AMGST employs an effective spatiotemporal embedding strategy that adaptively incorporates spatial and temporal information, enhancing the model’s ability to capture complex spatiotemporal correlations. By incorporating the spatiotemporal multi-head attention mechanism into the multi-graph diffusion convolution module, the model can extract long-term temporal dependencies and dynamic spatiotemporal relationships, thereby improving prediction accuracy.
3. The AMGST model is evaluated on four public traffic datasets for traffic flow and speed prediction tasks. Experimental results show that AMGST achieves strong performance compared to other baseline models, particularly in long-term forecasting.

The remainder of this paper is structured as follows. Section 2 reviews related work on traffic forecasting. In Section 3, we present the detailed architecture of the proposed AMGST method for traffic forecasting. Section 4 outlines the experimental setup and describes the datasets, evaluation metrics, and implementation details. Finally, Section 5 concludes the paper with a summary of findings, implications, and potential directions for future research.

## 2. Related work

### 2.1 Traffic prediction

In the field of traffic information prediction, traditional models such as the Kalman filter and Autoregressive Integrated Moving Average (ARIMA) are mainly used to process linear data. These models are characterized by low computational cost and simple structures. However, when applied to traffic data with multiple nodes, complex structures, and strong correlations, they struggle to adjust parameters in real time as the data distribution changes.

Machine learning methods such as decision trees, Naive Bayes, k-nearest neighbors (KNN), and support vector machines (SVM) can better handle parameter adjustments and achieve improved prediction accuracy. For example, Omar, M. et al. [14] proposed a Direct Particle Swarm Optimization Grid Search Support Vector Regression model (Direct PSOGS-SVR), which extends the SVR model to support multi-input and multi-output prediction, and uses particle swarm optimization and grid search to optimize hyperparameters. Luo, X. et al. [15] proposed a KNN-LSTM model, which uses KNN to select the nearest stations most related to the target station to capture spatial features, and then applies LSTM to extract temporal correlations from the selected stations. However, when dealing with large-scale and complex traffic data, regression-based and deep learning models tend to be more effective. These models are more effective at uncovering deeper spatiotemporal correlations within the data, thereby providing stronger support for traffic planning and decision-making.

In recent years, deep learning methods have also been widely used in traffic prediction, including recurrent neural networks (RNNs), long short-term memory networks (LSTM), and convolutional neural networks (CNN). CNN is particularly effective at mining spatial features and is widely used to extract spatial dependencies from traffic data. Yao, H. et al. [16] proposed the Deep Multi-View Spatiotemporal Network (DMVST-Net), which combines CNN and LSTM to model local spatial and temporal correlations. However, CNN-based traffic prediction methods often face performance degradation as network depth increases, which slows parameter updates and leads to a loss of correlation between local and global information, ultimately limiting prediction performance in practical applications.

The relationship between local and global data within a traffic network is crucial for accurate traffic information prediction and is naturally reflected in the graph structure of traffic network data. Effective extraction of this structure makes graph neural networks (GNNs) key to addressing traffic prediction challenges. For instance, DCRNN [17] applies the diffusion convolution concept from GNNs, replacing matrix multiplication in GRU units with diffusion graph convolution, thereby enhancing the model’s ability to capture spatial correlations. To further capture temporal dependencies and improve prediction accuracy, some models use enhanced RNNs based on GNNs to model time series data. However, RNNs are prone to gradient vanishing or explosion when handling long sequences. Consequently, other research efforts have combined GNNs with temporal convolutional networks (TCNs) to extract spatiotemporal features. Nevertheless, stacking convolution layers in long time series processing increases computational overhead.

Graph WaveNet addresses this by combining a modified TCN, dilated causal convolutions (DCNs), and GCNs, enabling the model to maintain a sufficient receptive field while using fewer layers to extract spatiotemporal characteristics. To further enhance the performance of graph convolution networks, Y. Sun et al. [18] proposed the Dual Dynamic Spatiotemporal Graph Convolutional Network (DDSTGCN), which transforms the traffic graph into a hypergraph to model both node and edge dynamics, thereby capturing spatiotemporal dependencies more comprehensively. Despite the strong performance of these spatiotemporal convolution models in local modeling, they still face challenges in achieving robust long-term prediction.

### 2.2 Attention mechanism

As an important computational approach in deep learning, the attention mechanism can dynamically focus on different representations of input data. It has been widely applied in various fields, including natural language processing, traffic flow prediction, and speech recognition [19–22]. Jiang et al. [23] introduced a multi-layer attention mechanism to model the dynamic spatiotemporal correlations between geographic sensors, aiming to address the problem of spatiotemporal data prediction. However, training a separate model for each time series can be time-consuming.

Wang et al. [24] proposed a spatiotemporal attention network that integrates attention mechanisms, dilated gated convolutions, and graph attention networks to construct multiple spatiotemporal blocks for traffic prediction. Additionally, Guo [25] proposed a Transformer-based spatiotemporal model for traffic flow prediction, which employs a newly designed attention mechanism to capture the temporal dynamics of traffic data and replaces the traditional feed-forward layer with graph convolution to extract spatial correlations. These models have demonstrated strong performance in traffic flow prediction tasks.

Beyond traffic flow forecasting, attention mechanisms have been widely applied in other domains. For example, Hu et al. [26] proposed M2BIST-SPNet for remaining useful life (RUL) prediction, in which a spatial–temporal attention-based convolutional network is introduced to effectively capture spatiotemporal dependencies, thereby achieving excellent long-term performance.

## 3. Proposed method

### 3.1 Problem definition

Given a directed weighted graph G= (V, E, A), where V is the set of nodes and E is the set of edges, and A is the adjacency matrix representing the graph. When modeling a traffic network as a directed weighted graph, V and E correspond to the set of sensors and the set of roads, respectively, and |V| = *N* denotes the number of sensors. The adjacency matrix $𝐀\in {\text{R}}^{N\times N}$ describes the relationships between nodes and typically determines edge weights based on connectivity or distance.

Within a time window of length [t+1,t+T], traffic data can be represented as ${𝒳}^{t+1:t+T}=\text{[}{𝐗}^{t+1},\text{\textbackslash{}hspace\{0.17em}\}{𝐗}^{t+2},\text{\textbackslash{}hspace\{0.17em}\ldots \}\;{𝐗}^{t+T}\text{]}\in {\text{R}}^{T\times N\times F}$, where ${𝐗}^{t+T}\in {\text{R}}^{N\times F}$ is denoted as the feature of nodes at time step T. This paper focuses on traffic flow and speed information and aims to predict future traffic flow and speed values. Given traffic data from the past $T^{\prime }$ time steps, denoted as ${𝐗}^{t-T^{\prime }+1:t}=\text{[}{𝐗}^{t+1},\text{\textbackslash{}hspace\{0.17em}\}{𝐗}^{t+2},\text{\textbackslash{}hspace\{0.17em}\ldots \}\;{𝐗}^{t+T}\text{]}\in {\text{R}}^{T\times N\times 1}$, the goal is to learn a mapping function f that maps historical data to future data. For a given graph G and historical traffic data, this mapping can be represented as:


$$\begin{array}{c} f\text{(}{𝐗}^{\;t-T^{\prime }+1:t},G\text{)}={𝐗}^{t+1:t+T} \end{array}$$ (1)

In this paper, we set T = T′ = 12, 12 steps of historical traffic flow data are used to predict the traffic flow for the next 12 steps.

### 3.2 AMGST prediction model

**Fig 1:**
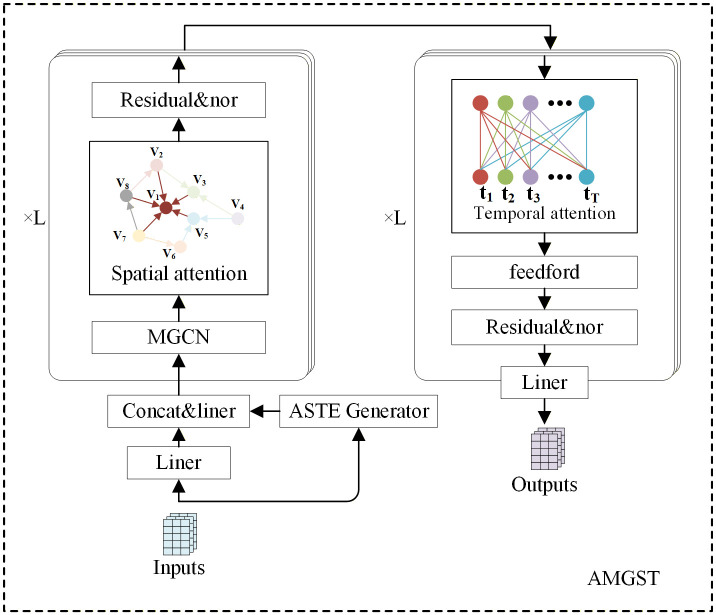
The overall framework of the AMGST.

Fig 1 illustrates the overall framework of the AMGST model, which includes adaptive embedding, graph convolutional layers, and spatiotemporal attention layers. The adaptive embedding merges the raw feature embedding, spatiotemporal adaptive embedding, and periodic embedding to produce features that incorporate spatiotemporal information. These features are then passed through multiple graph convolution layers, which utilize weighted adjacency matrices to learn spatial features. Finally, the spatiotemporal multi-head attention layer is applied to capture spatiotemporal dependencies.

The multi-graph convolution layer employs fine-grained adjacency matrices, enabling the learning of global spatial features. Multi-head self-attention is applied across both spatial and temporal dimensions. In the spatial dimension, attention dynamically allocates node weights, allowing for the adaptive update of spatial information. In the temporal dimension, multi-head self-attention directly captures long-range temporal dependencies—something that is difficult to achieve using temporal convolution (TCN). Residual connections and layer normalization are applied to both the convolution modules and the multi-head attention components at each layer. Finally, the prediction results are produced through the output layer.

### 3.3 ASTE generator

Most spatiotemporal graph neural networks (STGNNs) directly embed the input into the model through linear layers. Similarly, to extract the raw features from traffic data, this paper uses a linear layer to obtain the original feature embedding $E_{\text{data}}\in {\text{R}}^{B\times T\times N\times D_{da}}$,


$$E_{data}\;=\;linear\left(X_{t-T\;+1:t}\right)$$ (2)

In the above equation, *linear* denotes a linear layer, and $X_{(t-T+1):t}$ represents the input features $X\in {\text{R}}^{B\times T\times N\times F}$, The periodic embeddings consist of daily and weekly embeddings, denoted as $E_{d}\in {\text{R}}^{B\times T\times N\times D_{d}}$ and $E_{w}\in {\text{R}}^{B\times T\times N\times D_{w}}$, respectively. We initialize two trainable embedding matrices: $A_{D}\in {\text{R}}^{N_{d}\times D_{d}}$ and $A_{W}\in {\text{R}}^{N_{w}\times D_{w}}$, where $D_{d}$ and $\;D_{w}$ are their embedding dimensions. The number of traffic time series samples per day is represented by $N_{d}$ = 288 (since data is sampled every 5 minutes in the dataset used in this paper), and the number of days in a week is $N_{w}$ = 7. The input time sequence serves as an index into the embedding layers, and the final periodic embeddings $E_{d}$ and $E_{w}$ are obtained by extracting the corresponding entries from the embedding matrices.

Furthermore, this paper introduces adaptive spatiotemporal embeddings $E_{a}={\text{R}}^{B\times T\times N\times D_{a}}$, which are randomly initialized trainable parameters designed to capture dynamic spatiotemporal dependencies. These embeddings are learned directly during training, enabling the model to discover richer and more implicit spatiotemporal correlations in traffic flow data.

In summary, we concatenate these four embeddings to obtain the output of the embedding layer $E_{O}$, as shown in the following equation:


$$E_{O}=E_{data}\;\|E_{d}\;\;\|\;E_{w}\;\|\;\;E_{a}\in {\text{R}}^{B\times T\times N\times D}$$ (3)

Here, || denotes the concatenation operation, and the final embedding dimension is $D\;\;=\;\;D_{g}\;\;+\;\;D_{d}\;\;+\;\;D_{w}\;\;+\;\;D_{a}$. After training, $E_{O}$ contains rich spatiotemporal information, which helps the model better capture spatiotemporal correlations.

**Algorithm 1. ASTE generator.**

**Require**: Raw input $X\in {\text{R}}^{B\times T\times N\times F}$

 1: Hyperparameters: $D_{g},D_{d},D_{w},D_{a}$, $N_{d}$ = 288, $N_{w}$ = 7

 2: Initialize periodic lookup matrices $A_{D}\in {\text{R}}^{N_{d}\times D_{d}}$, $A_{W}\in {\text{R}}^{N_{w}\times D_{w}}$

 3: Initialize adaptive embedding parameter $\overset{\text{param}}{\underset{a}{E}}={\text{R}}^{T\times N\times D_{a}}$

 4: **Forward**:

 5: $E_{\text{data}}\leftarrow \text{Linear}(X;W_{\text{lin}},b_{\text{lin}})\text{\textbackslash{}hspace\{1em}\}\#shape\left(B,T,N,D_{data}\right)$

 6: $E_{w}\leftarrow \text{expand}(E_{w\_lookup},\text{\textbackslash{}hspace\{0.33em}to\}\_\text{nodes=N})\text{\textbackslash{}hspace\{0.33em}\;\;\;\}\#\left(B,T,N,D_{w}\right)$

 7: $E_{d}\leftarrow \text{expand}(E_{d\_lookup},\text{\textbackslash{}hspace\{0.33em}to\}\_\text{nodes=N})\text{\textbackslash{}hspace\{0.33em}\;\;\;\}\#\left(B,T,N,D_{d}\right)$

 8: $E_{a}\leftarrow \text{repeat}(\overset{\text{param}}{\underset{a}{E}},\text{\textbackslash{}hspace\{0.33em}repeat\}\_\text{batch=B})\text{\textbackslash{}hspace\{0.33em}\;\;\;\}\#expandto\left(B,T,N,D_{a}\right)$

 9: $E_{O}\leftarrow \text{Concat}\left(E_{\text{data}},E_{d},E_{w},E_{a}\right)\text{\textbackslash{}hspace\{0.33em}\;\;\;\}\#\left(B,T,N,D\right)$

 10: $W_{\text{lin}},b_{\text{lin}},A_{D},A_{W},\overset{\text{param}}{\underset{a}{E}}$ are all trainable parameters.

### 3.4 Graph convolutional layer

The graph convolution layer utilizes predefined matrices, including the adjacency matrix, maximum information coefficient (MIC) matrix, and a dynamic adaptive adjacency matrix, to extract spatial features. The following sections define these three matrices.

#### 3.4.1 Adjacency matrix.


$$\left\{ \begin{array}{c} @la_{ij}=1,if\;\;e_{ij}\in E\\ a_{ij}=0,others \end{array}\right.$$ (4)

In the formula, $e_{ij}\in E$ indicates that an edge exists in the edge set connecting the two nodes; otherwise, $a_{ij}$ is zero.

#### 3.4.2 Maximum mutual information coefficient matrix.

The maximal information coefficient (MIC) matrix $A_{\text{MIC}}$ measures the data correlation between nodes by calculating the MIC for each pair of nodes. The concept of MIC is to discretize two variables and represent their relationship as a scatter plot. In this process, the current two-dimensional space is divided into a certain number of intervals along the x-axis and y-axis, and then the distribution of scatter points in each grid cell is observed. For two sequences, if the y-axis is divided into a and b intervals, the MIC is calculated as follows:


$$MIC\left(X,Y\right)=MAX_{ab<B}\frac{I\left(X,Y\right)}{{log}_{\text{2}}min\left\{a,b\right\}}$$ (5)

Based on empirical experience, the parameter B is set to the total data size raised to the power of 0.6. The mutual information I (X, Y) can be calculated using the following equation:


$$I\left(X,Y\right)=\sum_{X<a,Y<b}p\left(X,Y\right){log}_{\text{2}}\frac{p\left(X,Y\right)}{p\left(X\right)p\left(Y\right)}$$ (6)

where p(X) and p(Y) are the probabilities of the respective values falling with their intervals, and p (X, Y) is the joint probability of both variables. In this paper, MIC is calculated for all features to better explore spatial correlations between nodes. If $\overset{F}{\underset{i}{X}}$ is the sequence of sensor i for feature F, then the element $a_{ij}$ in the maximal information coefficient matrix $A_{\text{MIC}}$ can be given by the following formula:


$$\overset{F}{\underset{ij}{a}}=MIC\left(\overset{F}{\underset{i}{X}},\overset{F}{\underset{j}{X}}\right)$$ (7)

where $A_{\text{MIC}}\in {\text{R}}^{N\times N\times F}$, in graph convolution, we weight each feature into a matrix and finally feed it into the diffusion graph convolutional network. $A_{\text{MIC}}$ is a fine-grained similarity representation, where the elements range from 0 to 1. Values closer to 1 indicate a higher similarity in traffic patterns and stronger correlation between nodes, while values closer to 0 indicate significant differences and weaker correlation. In this work, $A_{\text{MIC}}$ is computed by calculating MIC for every pair of traffic flow time series between nodes across the entire training period. The resulting matrix is symmetric, as the MIC between node i and node j is identical to that between node j and node i. Each element is normalized to lie between 0 and 1, and no additional thresholding is applied. In multi-feature scenarios, MIC is computed independently for each feature, producing a tensor of shape ${\text{R}}^{N\times N\times F}$, where N is the number of nodes and F is the number of features. The MIC matrix is precomputed and fixed during training, which ensures that it does not introduce additional computational overhead in the model training or inference process.is a fine-grained similarity representation, where the elements range from 0 to 1. Values closer to 1 indicate a higher similarity in traffic patterns and stronger correlation between nodes, while values closer to 0 indicate significant differences and weaker correlation.

#### 3.4.3 Adaptive adjacency matrix.

To further capture latent spatial correlations, we construct two learnable embedding matrices, $W_{\text{1}}$ and $W_{\text{2}}$ $\in {\text{R}}^{N\times C_{\text{emb}}}$, where $C_{\text{emb}}$ is the embedding dimension. Through training, both $W_{\text{1}}$ and $W_{\text{2}}$ can learn certain spatial features. The calculation of the adaptive weighted adjacency matrix is as follows:


$$E_{\text{1}}=\text{Tanh}\left(\alpha W_{\text{1}}\right)\;$$ (8)

$$E_{\text{2}}=\text{Tanh}\left(\alpha W_{\text{2}}\right)$$ (9)

$$A_{adp}=sofmax(relu\left(\alpha \left(E_{\text{1}}{E_{\text{2}}}^{T}-{E_{\text{1}}}^{T}E_{\text{2}}\right)\right)$$ (10)

where α is a parameter controlling the saturation of the activation function, and $E_{\text{1}}$, $E_{\text{2}}\in {\mathbb{R}}^{N\times C_{\text{emb}}}$ are intermediate variable matrices used to generate the adaptive adjacency matrix $A_{\text{adp}}$; in this paper, $W_{\text{1}}$ and $W_{\text{2}}$ are randomly initialized.

#### 3.4.4 Multi-graph diffusion convolution.

Before performing graph convolution, the adjacency matrix is often normalized to prevent erroneous learning during the convolution process. Common normalization methods include the symmetric normalized Laplacian matrix and the random walk matrix.

First, we need to compute the degree matrix D, which is a diagonal matrix with diagonal elements $D_{ii}$ equal to the in-degree of each row of the adjacency matrix, i.e., $D_{ii}=\text{Rowsum}\left(A_{i}\right)$. The Laplacian matrix is defined as L = D-A.

The random walk matrix is defined as follows, where each element represents the transition probability from one node to another:


$$A^{r}=D^{-1}A$$ (11)

Let the normalized adjacency matrix be $\tilde{A}\in {\text{R}}^{N\times N}$, the input feature matrix $X\in {\text{R}}^{N\times D}$, and the learnable weight matrix $W\in {\mathbb{R}}^{D\times M}$. The graph convolution can be expressed as:


$$Z=\tilde{A}XW$$ (12)

Diffusion graph convolution models spatial correlations by combining traffic flow with a diffusion process, described in the form of random walk matrices and extended to directed graphs. The forward propagation matrix is $P_{f}=A^{r}$ and the backward propagation matrix is $P_{b}=(A^{r}{)}^{T}$. Therefore, to better model traffic flow, the diffusion graph convolution is defined as follows:


$$Z=\sum_{K=0}^{K}\overset{k}{\underset{f}{P}}XW_{k1}+\overset{k}{\underset{b}{P}}XW_{k2}$$ (13)

The adaptive adjacency matrix $A_{\text{adp}}$ and $A_{\text{MIC}}$ are combined to explore more fine-grained spatial correlations. Using second-order diffusion convolution (K = 2), local spatial information is mined. The final graph convolution formula is given as follows:


$$Z=\sum_{K=0}^{K}\overset{k}{\underset{f}{P}}XW_{k1}+\overset{k}{\underset{b}{P}}XW_{k2}+\overset{k}{\underset{adp}{A}}XW_{k3}+{\overset{k}{\underset{adp}{A}}}^{T}XW_{k4}+\overset{k}{\underset{mic}{A}}XW_{k5}$$ (14)

### 3.5 Spatial and temporal attention layer

The self-attention mechanism was originally used in natural language processing to explore correlations between words. The inputs Q, K, and V are the values of the attention layer after feature mapping. The multi-head attention mechanism contains multiple independent learnable weight matrices Q, K, and V, which are independently initialized randomly. These matrices map the input vectors into different subspaces, enabling the model to capture richer features and more diverse patterns from the input data.

In this paper, we apply the multi-head self-attention mechanism to both the temporal and spatial axes of the input data. The spatial self-attention mechanism is defined as follows:

First, from the input $Z\in {\text{R}}^{T\times N\times D}$, obtain $Q^{\left(s\right)}$, $K^{\left(s\right)}$, and $V^{\left(s\right)}$ through mapping as follows:


$$\;Q^{\left(s\right)}=Z\overset{\left(s\right)}{\underset{Q}{W}},K^{\left(s\right)}=Z\overset{\left(s\right)}{\underset{K}{W}},V^{\left(t\right)}=Z\overset{\left(s\right)}{\underset{V}{W}}$$ (15)

where $Q^{\left(s\right)}$, $K^{\left(s\right)}$, and $V^{\left(s\right)}\in {\text{R}}^{T\times N\times D_{s}}$, $D_{s}$ is the dimension of the mapping. $\overset{\left(s\right)}{\underset{Q}{W}}$, $\overset{\left(s\right)}{\underset{K}{W}}$, $\overset{\left(s\right)}{\underset{V}{W}}\in {\text{R}}^{D\times D_{s}}$ are learnable parameter. The calculation formula for the spatial attention scores is as follows:


$$A^{\left(s\right)}=Softmax\left(\frac{Q^{\left(s\right)}K^{\left(s\right)T}}{\sqrt{D_{s}}}\right)$$ (16)

The spatial attention scores $A^{\left(s\right)}\in {\text{R}}^{N\times T\times T}$ is then multiplied by the value matrix $V^{\left(s\right)}$ to yield the final temporal attention output:


$$Z^{\left(s\right)}=A^{\left(s\right)}V^{\left(s\right)}$$ (17)

We use the temporal attention mechanism to update the temporal features of nodes. To capture richer information, we extend it into a multi-head self-attention mechanism: First, the input is mapped into H groups of $Q^{\left(s\right)}$, $K^{\left(s\right)}$, and $V^{\left(s\right)}$, obtaining H parallel temporal attention outputs. These outputs are then concatenated to produce the result:


$$MZ^{\left(s\right)}=[{Z^{\left(s\right)}}_{\text{1}};{Z^{\left(s\right)}}_{\text{2}};\ldots ;{Z^{\left(s\right)}}_{H}]\overset{\left(s\right)}{\underset{O}{W}}$$ (18)

In the formula, $\overset{\left(s\right)}{\underset{O}{W}}\in {\mathbb{R}}^{D\times hD_{t}}$ is learnable parameter, $D_{s}$ will be set to D/H. The temporal self-attention mechanism follows a similar structure to the spatial attention. From equations (15)–(17), the temporal attention SelfAttention() is obtained, and its calculation is given as:


$$Z^{\left(t\right)}=SelfAttention\left(Z^{\left(s\right)}\right)$$ (19)

Similar to spatial attention, temporal attention also employs a multi-head mechanism to enhance its expressive capability.


$$MZ^{\left(t\right)}=\left[{Z^{\left(t\right)}}_{\text{1}};{Z^{\left(t\right)}}_{\text{2}};\ldots ;{Z^{\left(t\right)}}_{H}\right]\overset{\left(t\right)}{\underset{O}{W}}$$ (20)

The self-attention mechanism allows each data representation to be directly influenced by all other data in the sequence, enabling the model to achieve a global receptive field. Therefore, the proposed model can reveal hidden long-range patterns and capture global dependencies beyond adjacent nodes. Temporal attention, in particular, enables distant dependencies to be processed in parallel, benefiting model training. Moreover, it can be directly extended to sequences spanning multiple time steps without increasing the model’s depth and complexity.

Moreover, residual connections and layer normalization are applied after each attention module to stabilize learning. Finally, the extracted spatiotemporal information is first passed through a fully connected layer, and ultimately outputs the final prediction through a Leaky ReLU activation function and a linear transformation layer.

## 4. Experiments

This section first introduces the datasets, baseline methods, and evaluation metrics used in this paper. We conduct analysis and prediction of traffic flow and speed on four traffic datasets: PEMS04, PEMS08, PEMS-BAY, and METR-LA, and compare the results with eight baseline methods. Finally, we perform ablation studies and parameter analysis on the proposed AMGST model. All experiments in this paper are conducted on a GPU equipped with a GeForce RTX 4060 (8GB memory) within the PyTorch programming environment.

### 4.1 Datasets

This paper conducts experiments on four datasets from the Performance Measurement System (PEMS): PEMS04, PEMS08, METR-LA, and PEMS-BAY. PEMS04 contains flow data collected by 307 detectors over 59 consecutive days, starting from January 1, 2018. PEMS08 provides flow data from 170 nodes over 62 consecutive days, beginning on July 1, 2016. The METR-LA dataset includes four months of traffic speed statistics collected from 207 sensors on highways in Los Angeles County. In contrast, the PEMS-BAY dataset contains six months of traffic speed data collected from 325 sensors in the Bay Area. The collection interval for both traffic flow and speed data is five minutes. Detailed information is shown in Table 1. For all datasets, we use traffic data from the past hour to predict traffic data for the next hour.

**Table 1:** Dataset details.

Datasets	Date	Nodes	Time steps	Period	Target
PEMS04	2018/01/01–2018/02/28	307	16,992	5 min	Flow
PEMS08	2016/07/01–2016/08/31	170	17,856	5 min	Flow
METR-LA	2012/03/01-2012/06/30	207	34272	5 min	Speed
PEMS-BAY	2017/01/01-2017/05/31	325	52116	5 min	Speed

### 4.2 Baselines & evaluation metrics

We compare the performance of proposed AMGST model with the following 9 models.

**DCRNN** [17]: Diffusion Convolutional Recurrent Neural Network. This model combines diffusion convolution and RNN, using a bidirectional random walk matrix to model spatial dependencies and RNN to capture temporal dependencies by remembering previous states.

**STGCN** [3]: Spatiotemporal Graph Convolutional Network. A deep learning model that integrates gated temporal convolution and graph convolution to extract multi-level spatiotemporal features.

**Graph aveNet** [5]: A model based on WaveNet and graph convolutional networks. It employs an adaptive adjacency matrix to fully utilize both graph structural information and time series data.

**AGCRN** [27]: Adaptive Graph Convolutional Recurrent Network. This model combines adaptive graph convolution with recurrent neural networks (RNN) to capture dynamic spatial-temporal dependencies.

**DGCRN** [28]: Dynamic Graph Convolutional Recurrent Network. A deep learning model based on GCN and RNN that dynamically updates the adjacency matrix during training.

**STID** [13]: Spatial-Temporal Identity. A simple linear model that uses embedding and linear layers to capture spatiotemporal patterns.

**GMAN** [8]: Graph Multi-Attention Network. An attention-based model that integrates spatial attention, temporal attention, and transformation attention for traffic prediction.

**DDSTGCN** [18]: Dual Dynamic Spatial-Temporal Graph Convolution Network for Traffic Prediction. A deep learning framework that integrates TCN and GCN, and transforms the traffic graph into a hypergraph to extract complex spatiotemporal relationships.

**MTGNN** [29]: Multivariate Time Series Forecasting with Graph Neural Networks. This framework uses a mix-hop propagation graph convolution module and dilated causal convolution layers to capture both spatial and temporal dependencies in time series data.

To evaluate the performance of traffic information prediction models, we adopt three commonly used metrics: Mean Absolute Error (MAE), Mean Absolute Percentage Error (MAPE), and Root Mean Square Error (RMSE). The specific expressions are as follows:


$$MAE=\frac{\text{1}}{n}\sum_{i=1}^{N}\left|y_{i}-\hat{y_{i}}\right|$$ (21)

$$MAPE=\frac{\text{1}}{n}\left(\sum_{i=1}^{n}\left|\frac{y_{i}-\hat{y_{i}}}{y_{i}}\right|\right)$$ (22)

$$RMSE=\sqrt{\frac{\text{1}}{n}\sum_{i=1}^{n}{\left|y_{i}-\hat{y_{i}}\right|}^{\text{2}}}$$ (23)

where $y_{i}$ and $\hat{y_{i}}$ represent the true and predicted values of traffic flow at the time step, respectively. MAE, MAPE and RMSE reflect the difference between the predicted value and the true value. Smaller values for these metrics indicate better performance.

During the calculation of MAPE, samples with zero ground-truth values are masked out to avoid division-by-zero issues. According to the zero-value proportions summarized in Table 2, all four datasets contain less than 8% zero values, indicating that MAPE remains a stable and valid evaluation metric for these datasets.

**Table 2:** Dataset details.

Datasets	Zero-value proportion (%)	Zero count/ Total count
PEMS04	5.73%	896792/15649632
PEMS08	5.07%	461740/9106560
METR-LA	7.58%	2492946/50813100
PEMS-BAY	4.91%	1613407/21282912

### 4.3 Parameter setting

For the PEMS04 and PEMS08 datasets used in the experiments, the data are split into training, validation, and test sets in a 6:2:2 ratio. For the PEMS-BAY and METR-LA datasets, we follow the data split setting of DCRNN, using a 7:1:2 ratio for training, validation, and testing. The comparative experimental results are also aligned with the DCRNN setup, presenting speed prediction performance for 15, 30, and 60-minute horizons. Before being fed into the model, the input data are standardized by removing the mean and scaling to the standard deviation, as shown below, where μ and σ denote the mean and standard deviation of the traffic flow series, respectively.


X=σ(X−μ) (24)

In the AMGST model, the dimensions of the periodic embedding and spatiotemporal embedding are set to 48 and 40, respectively. In the MIC matrix, the parameters α and c are set to 0.6 and 15, respectively. The batch size is set to 16, the learning rate to 0.001, the maximum number of iterations to 200, and the early stopping patience to 20 epochs. The optimizer used is Adam, with a weight decay of 0.0005. Detailed parameter settings are shown in Table 3. In this experiment, both the input and output sequence lengths are set to 12, corresponding to predicting traffic flow or speed for the next hour. Additionally, an early stopping strategy is adopted, where training is halted if the validation loss does not improve for 20 consecutive epochs. Except for the batch size, all other experimental settings of the baseline models are kept consistent with those in their original papers.

**Table 3:** Parameter settings.

	hyper-parameters	Setup
Model	$D_{d}+D_{w}$	48
	$D_{a}$	40
	$D_{data}$	24
	Dropout	0.1
	batch size	16
	Feed-forward dim	128

### 4.4 Comparative experimental results

Table 4 presents the experimental results of different methods on the METR-LA and PEMS-BAY datasets, reporting three error metrics for traffic flow forecasting at 15-minute, 30-minute, and 60-minute horizons. At the 60-minute horizon, AMGST achieves lower error values across all three metrics on both datasets compared with the baseline models. In contrast, at the 15-minute horizon, its performance is slightly weaker than that of DGCRN and DDSTGCN on METR-LA, and DDSTGCN and STID on PEMS-BAY. Nevertheless, as the prediction horizon increases, AMGST consistently demonstrates more competitive performance and yields lower prediction errors overall, highlighting its effectiveness in capturing long-term spatiotemporal dependencies in traffic data.

**Table 4:** Performance comparison of different benchmark models on METR-LA and PEMS-BAY datasets.

Datasets	Models	15min(3step)			30min(6step)			60(12step)		
		MAE	RMSE	MAPE(%)	MAE	RMSE	MAPE(%)	MAE	RMSE	MAPE(%)
META-LA	DCRNN	2.67	5.17	6.84	3.07	6.29	8.39	3.57	7.52	10.34
	STGCN	2.74	5.29	7.02	3.13	6.33	8.42	3.58	7.41	10.06
	GWNET	2.69	5.15	6.89	3.09	6.19	8.33	3.55	7.31	9.91
	AGCRN	2.85	5.53	7.72	3.22	6.55	9.12	3.60	7.50	10.62
	DGCRN	**2.61**	**4.98**	**6.67**	2.98	6.04	**8.03**	3.45	7.28	10.93
	DDSTGCN	2.64	5.01	6.76	3.00	**6.02**	8.12	3.44	7.13	9.74
	STID	2.80	5.52	7.73	3.17	6.58	9.34	3.54	7.53	9.77
	GMAN	2.80	5.55	7.41	3.12	6.49	8.73	3.44	7.35	10.07
	MTGNN	2.69	5.20	6.92	3.05	6.22	8.27	3.49	7.32	9.88
	AMGST	2.64	5.06	6.85	**2.96**	6.03	8.08	**3.34**	**7.05**	**9.59**
PEMS-BAY	DCRNN	1.31	2.75	2.72	1.65	3.76	3.71	1.97	4.59	4.69
	STGCN	1.35	2.82	2.85	1.70	3.79	3.83	2.02	4.60	4.78
	GWNET	1.31	2.76	2.73	1.66	3.80	3.73	2.00	4.64	4.65
	AGCRN	1.34	2.83	2.90	1.66	3.76	3.80	1.93	4.47	4.56
	DGCRN	1.31	2.73	2.73	1.63	3.68	3.65	1.94	4.46	4.59
	DDSTGCN	**1.29**	**2.71**	**2.69**	**1.60**	**3.64**	**3.62**	**1.89**	4.37	4.46
	STID	1.30	2.77	2.75	1.62	3.70	3.71	1.90	4.38	4.51
	GMAN	1.35	2.90	2.87	1.65	3.82	3.74	1.92	4.49	4.52
	MTGNN	1.33	2.82	2.81	1.65	3.78	3.71	1.95	4.52	4.53
	AMGST	1.32	2.78	2.78	1.61	3.68	3.65	**1.89**	**4.30**	**4.40**

Table 5 shows the average performance over 12 time steps of different methods on the PEMS04 and PEMS08 datasets. As shown in Table 5, AMGST exhibits a clear advantage on both PEMS04 and PEMS08, outperforming all other baseline methods. Overall, AMGST demonstrates excellent performance on both speed and flow datasets, validating its generalization ability and effectiveness.

**Table 5:** Performance comparison of different benchmark models on the PEMS04 and PEMS08 datasets.

Models	PEMS04			PEMS08		
	MAE	RMSE	MAPE(%)	MAE	RMSE	MAPE(%)
DCRNN	20.73	33.23	14.61	16.39	25.27	14.02
STGCN	20.07	31.89	13.83	16.07	27.83	11.40
Gragh wavenet	19.52	30.87	14.57	15.01	23.57	10.00
AGCRN	19.40	31.32	13.26	15.77	24.70	10.29
DGCRN	18.84	30.57	13.42	14.68	23.52	9.51
DDSTGCN	19.99	31.47	14.54	15.64	24.54	11.28
STID	18.55	30.20	12.16	14.29	23.59	9.25
GMAN	18.83	30.93	13.21	14.81	24.19	9.69
MTGNN	19.40	31.32	13.23	15.20	24.18	9.79
AMGST	**18.49**	**30.12**	**12.08**	**13.66**	**23.09**	**9.09**

Among the baseline models, the predictive performance of DCRNN and AGCRN is constrained by the limitations of RNNs, making them weaker than most spatiotemporal graph convolution methods. On the PEMS08 dataset, AMGST reduces MAE, RMSE, and MAPE by 10.8%, 9.4%, and 17.3%, respectively, compared to DCRNN. Both STGCN and DDSTGCN are static spatiotemporal graph convolution methods. DDSTGCN improves performance over traditional static graphs by converting the static adjacency matrix into a hypergraph convolution to learn the spatial correlations of the edge set, yet it still falls short by neglecting long-range spatial dependencies. On the PEMS04 and PEMS08 datasets, AMGST outperforms STGCN and DDSTGCN with MAE, RMSE, and MAPE reductions of 7.9%, 5.6%, 12.7% and 14.8%, 16%, 20.3%, respectively. Compared to DDSTGCN, AMGST achieves reductions of 7.5%, 4.3%, 16.9% and 12.5%, 4.7%, 19.4%, respectively. Graph WaveNet and MTGNN outperform the previously mentioned spatiotemporal graph models by leveraging adaptive graph matrices in graph convolution networks. DGCRN dynamically generates node features based on current speed, temporal encoding, and previous hidden states. However, its adaptive graph structure fails to fully represent similar patterns among different sensors. By computing a maximum mutual information matrix, the proposed AMGST captures broader node connections and learns more accurate spatial similarity features through the graph convolution module. For attention-based models, GMAN improves performance to some extent by extracting spatial and temporal dependencies separately via multi-head self-attention and fusing them with a gating mechanism. With multilayer spatiotemporal attention, AMGST can better extract temporal features and aggregate spatiotemporal correlations. Compared to GMAN, AMGST improves MAE, RMSE, and MAPE by 7.56% on the PEMS04 and PEMS08 datasets. In comparison to Graph WaveNet, AMGST achieves improvements of 5.3%, 2.4%, 17.1%, 8.8%, 0.8%, 9.1%, 3.9%, 2.6%, 3.7%, and 2.5%, 4.6%, 2.5% on the PEMS04, PEMS08, METR-LA, and PEMS-BAY datasets, respectively.

**Fig 2:**
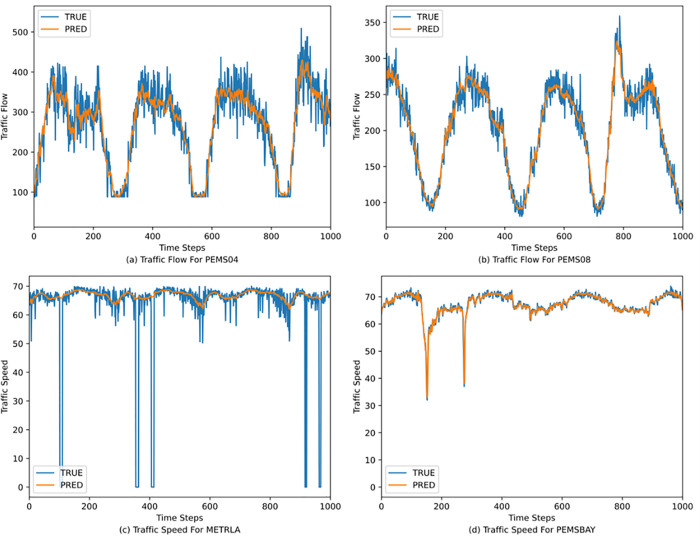
Prediction results of random nodes on four datasets at continuous step sizes.

To visually analyze the prediction performance of the AMGST model, this paper randomly selects one sensor node from each of the four datasets and presents their predicted values and true values over 1,000 consecutive time steps. The prediction results are shown in Fig 2. It is clearly observed from Fig 2 that AMGST effectively captures the spatiotemporal features of the data, fitting the changing trends of traffic flow and speed well. Traffic flow and speed exhibit obvious temporal and spatial characteristics, typically showing sudden increases in flow and sharp decreases in speed during morning and evening peak hours. AMGST is able to identify such abrupt changes in traffic flow and speed to a certain extent, producing relatively accurate predictions. The multi-head attention mechanisms in both temporal and spatial dimensions help extract long-term temporal dependencies and dynamic spatiotemporal correlations of traffic flow and speed, enabling AMGST to fit large fluctuations in traffic information and achieve strong predictive performance.

**Fig 3:**
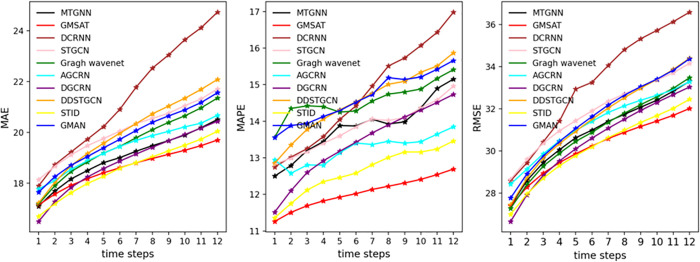
Prediction results on PEMS04.

**Fig 4:**
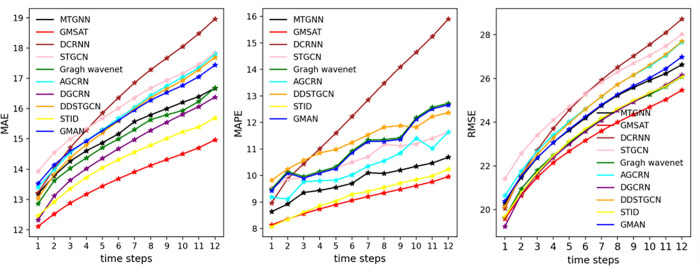
Prediction results on PEMS08.

To analyze the prediction trend of the proposed AMGST model compared to other baseline methods on the PEMS04 and PEMS08 datasets within one hour, further validating the effectiveness and long-term performance of AMGST, Figs 3 and 4 present the prediction results over 12 time steps. Considering the 12-step prediction curves across all four datasets, the MAE, MAPE, and RMSE curves of the AMGST model are the most stable. Its prediction results outperform most baselines at most time steps, especially between the 6th and 12th steps, demonstrating superior performance in mid-term forecasting. This indicates that our model effectively captures long-term temporal dependencies. Among all models, AMGST’s 12-step prediction curves are the smoothest, and its performance degrades the slowest as the time steps increase. In contrast, models like DCRNN and ST_AE show a significant performance decline with longer prediction horizons. STID also experiences some performance degradation over time, ranking just below AMGST overall across the four datasets, but with notably larger performance loss on the PEMS04 dataset, indicating weaker long-term forecasting ability compared to AMGST.

We conducted statistical and significance analyses of AMGST compared with the two strong baseline models, STID and DDSTGCN, as shown in Table 6. On the PEMS04 dataset, AMGST significantly outperformed DDSTGCN on MAE (t = −11.43, p = 0.0002), RMSE (t = −9.74, p = 0.0005), and MAPE (t = −15.25, p = 0.0001). In contrast, there were no statistically significant differences between AMGST and STID for any metric. Therefore, on PEMS04, AMGST is clearly superior to DDSTGCN while comparable to STID. On the METR-LA dataset, no significant differences were observed between AMGST and DDSTGCN across the evaluated metrics, whereas AMGST significantly outperformed STID on all metrics. This indicates that on METR-LA, AMGST performs similarly to DDSTGCN but substantially better than STID.

**Table 6:** Statistical and significance analysis of AMGST.

Dataset	Metric	AMGST	DDSTGCN	STID	AMGST-DDSTGCN (t, p)	AMGST-STID (t, p)	DDSTGCN-STID (t, p)
PEMS04	MAE	18.505 ± 0.038	19.793 ± 0.191	18.536 ± 0.016	(−11.43, 0.0002)	(−0.70,0.538)	(10.96, 0.0003)
	RMSE	30.237 ± 0.141	31.250 ± 0.210	30.182 ± 0.022	(−9.74, 0.0005)	(−0.08, 0.939)	(10.18, 0.0004)
	MAPE	12.107 ± 0.024	14.060 ± 0.424	12.153 ± 0.006	(−15.25,0.0001)	(−0.45,0.678)	(14.90, 0.0001)
METR-LA	MAE	2.945 ± 0.018	2.970 ± 0.010	3.127 ± 0.015	(−1.25, 0.278)	(−21.50,0.0001)	(−20.60, 0.0001)
	RMSE	6.000 ± 0.053	5.953 ± 0.045	6.497 ± 0.035	(0.83,0.449)	(−20.10,0.0001)	(−20.50, 0.0001)
	MAPE	7.979 ± 0.038	8.027 ± 0.076	9.157 ± 0.050	(−0.59,0.592)	(−21.40,0.0001)	(−20.80, 0.0001)

Overall, AMGST demonstrates strong performance and competitiveness on both PEMS04 and METR-LA datasets.

### 4.5 Ablation experiment

To verify the effectiveness of the model, this section conducts ablation experiments using the following four variants of the AMGST model:

AMGST/ $E_{a}$: Removes the adaptive embedding, using only the original feature embedding and temporal embedding.
AMGST/ $A_{MIC}$: Removes the MIC matrix from the multi-graph convolution module, using only the adaptive matrix.
AMGST/ $A_{adp}$: Removes the adaptive matrix. from the multi-graph convolution module, using only the MIC matrix.
AMGST/T: Removes the temporal attention in the temporal layer.
AMGST/S: Removes the spatial attention in the spatial attention layer.
AMGST/ST: Simultaneously removes the spatiotemporal attention mechanism.

From Table 7, it can be seen that removing the adaptive embedding leads to a significant decline in prediction performance across all datasets. The proposed model, which integrates the original feature embedding and temporal embedding, effectively represents the input data in both spatial and temporal dimensions. Removing the adaptive matrix from the multi-graph convolution module while using only the MIC matrix results in slightly worse performance than the full model, indicating that the adaptive matrix contributes complementary spatial information that helps capture latent correlations beyond what the MIC matrix alone can provide. After removing the temporal attention layer, the average MAE across the four datasets increases by approximately 2.5% compared to AMGST, highlighting the importance of modeling temporal dependencies. Removing the spatial attention layer also slightly degrades performance, whereas removing both temporal and spatial attention simultaneously (AMGST/ST) causes a substantial increase in MAE—about 25.5% on average—demonstrating that the spatiotemporal attention mechanism is critical for learning complete dependencies and complex spatiotemporal correlations. Overall, these results indicate that each component of AMGST—the adaptive embedding, the adaptive and MIC matrices in multi-graph convolution, and the spatiotemporal attention mechanism—plays an important role in capturing spatial and temporal patterns, and their combination leads to the most accurate traffic flow predictions.

**Table 7:** Experimental results of four variants.

Datasets	Model	MAE	RMSE	MAPE(%)
PEMS04	AMGST/ $E_{a}$	19.57	31.32	13.22
	AMGST/ $A_{MIC}$	18.71	30.82	12.34
	AMGST/T	18.89	31.07	12.19
	AMGST/S	18.78	30.97	12.15
	AMGST/ST	23.03	38.99	16.03
	AMGST	**18.49**	**30.12**	**12.08**
PEMS08	AMGST/ $E_{a}$	13.92	23.57	9.33
	AMGST/ $A_{MIC}$	13.97	23.45	9.35
	AMGST/T	13.96	23.47	9.33
	AMGST/S	14.11	23.41	9.50
	AMGST/ST	17.95	28.69	12.46
	AMGST	**13.69**	**23.39**	**9.09**
METR-LA	AMGST/ $E_{a}$	3.30	6.69	9.26
	AMGST/ $A_{MIC}$	2.98	6.09	8.12
	AMGST/T	2.99	6.13	8.16
	AMGST/S	2.98	6.11	8.14
	AMGST/ST	4.57	8.76	13.16
	AMGST	**2.94**	**6.00**	**7.99**
PEMS-BAY	AMGST/ $E_{a}$	1.69	3.86	3.80
	AMGST/ $A_{MIC}$	1.65	3.73	3.63
	AMGST/T	1.62	3.62	3.55
	AMGST/S	1.63	3.66	3.59
	AMGST/ST	2.01	4.24	4.55
	AMGST	**1.54**	**3.56**	**3.50**

### 4.6 Computation cost

We compared the per-epoch training time and inference time of AMGST with several baseline models on the PEMS08 dataset, as shown in Table 8. All models were trained with a batch size of 16. As can be observed, the computational time of AMGST is second only to DDSTGCN, while its performance surpasses that of other representative models. DDSTGCN is a typical spatiotemporal convolutional model; although it requires less computation time, its performance on PEMS08 is inferior. GMAN, which is based on the attention mechanism and employs an encoder–decoder architecture, exhibits higher computational cost than other compared models. DCRNN and DGCRN both rely on recurrent neural networks to capture temporal dependencies, which also results in higher computational time than AMGST.

**Table 8:** Computation time of the PEMS08 dataset.

Models	Computation time	
	Training(s/epoch)	Inference/s
DCRNN	121.3	12.5
GMAN	210.6	14.7
DDSTGCN	60.2	4.5
DGCRN	142.7	5.3
AMGST	86.8	5.8

### 4.7 Parameter study

This subsection analyzes the model performance under different configurations, such as varying numbers of attention heads, dropout values, attention layer depths, and diffusion convolution orders K. Table 9 presents the results of a sensitivity study where the number of attention heads is set to 1, 2, 4, 8, and 16 across all four datasets.

**Table 9:** Sensitivity study results of different attention heads.

Datasets	Att_heads	MAE	RMSE	MAPE(%)
PEMS04	1	18.53	30.24	12.13
	2	18.60	30.18	12.04
	**4**	**18.49**	30.12	12.08
	8	**18.49**	**30.10**	**12.02**
	16	18.57	30.20	12.08
PEMS08	1	13.83	23.33	9.21
	2	13.69	23.39	9.09
	**4**	13.91	23.57	9.32
	8	13.79	23.33	9.22
	16	**13.64**	**23.28**	**9.03**
METR-LA	1	2.97	6.08	8.12
	2	2.95	6.01	8.10
	**4**	**2.94**	**6.00**	**7.99**
	8	2.95	6.01	8.01
	16	**2.94**	**6.00**	8.00
PEMS-BAY	1	1.55	3.55	3.46
	2	1.55	**3.54**	**3.45**
	**4**	**1.54**	3.56	3.50
	8	1.55	3.56	3.45
	16	1.54	3.54	3.45

Table 9 clearly shows that increasing the number of attention heads enhances the model’s learning capacity. As the number of attention heads grows, both model complexity and accuracy improve. However, too many attention heads may lead to overfitting, causing a decline in prediction performance. The model achieves the best accuracy on METR-LA and PEMS-BAY datasets when using 4 attention heads, so the number of attention heads for these datasets is set to 4. Although PEMS04 reaches its best performance with 8 attention heads, the performance with 4 heads is comparable. Given this, and to balance model complexity, the number of attention heads for PEMS04 is set to 4. Similarly, to reduce computational overhead, the number of attention heads for PEMS08 is set to 2.

**Fig 5:**
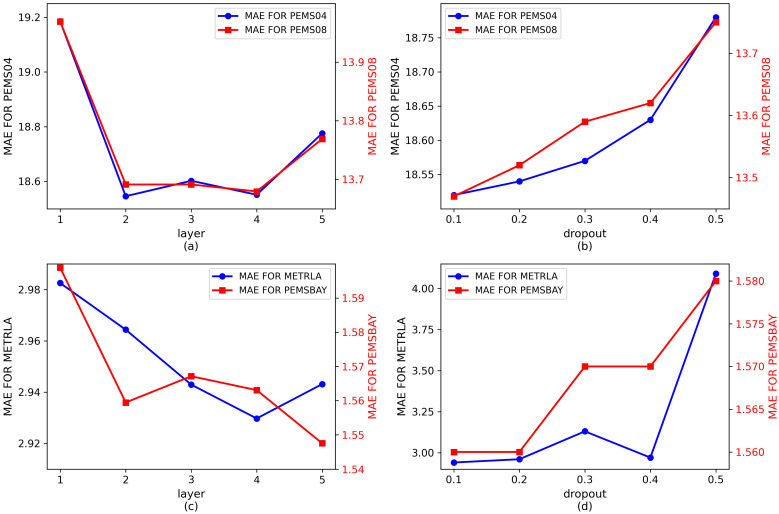
MAE changes of different attention levels and dropout values on all datasets.

Fig 5 presents the sensitivity analysis of different numbers of attention layers and dropout values across all datasets. As shown in Fig 5(b) and 5(d), applying dropout during feature propagation discards a certain proportion of feature values, which helps improve the model’s generalization ability. However, as dropout increases, more information is lost, leading to performance degradation. Therefore, dropout rate of 0.1 is used for all four datasets. Meanwhile, Fig 5(a) and 5(c) illustrate that stacking multiple self-attention layers enables the model to learn long-term spatiotemporal dependencies. By adjusting the number of layers to an optimal level, performance is enhanced while overfitting is avoided. Fig 5(a) and 5(c) shows that PEMS04 and PEMS08 achieve their best results with 2 layers, and increasing layers beyond that does not significantly enhance performance. Hence, the number of layers for PEMS04 and PEMS08 is set to 2. Similarly, Fig 5(c) depicts the trend of MAE for METR-LA and PEMS-BAY as the number of layers changes, leading to the conclusion that the layer counts are set to 4 and 5, respectively, for these datasets.

Table 10 presents the performance of AMGST on the PEMS04 and METR-LA datasets as the value of K varies from 1 to 3. It can be observed that as K increases, the prediction error first decreases significantly and then becomes stable. Considering that a larger K increases the model’s learning complexity, K is set to 2 in this study.

**Table 10:** Performance comparison under different K values.

Datasets	K	MAE	RMSE	MAPE (%)
PEMS04	1	19.18	30.57	13.64
	2	18.49	**30.12**	**12.08**
	3	**18.48**	30.19	12.12
METR-LA	1	3.10	6.47	8.78
	2	2.94	6.00	7.99
	3	**2.93**	**5.94**	**7.93**

### 4.8 Visualization

**Fig 6:**
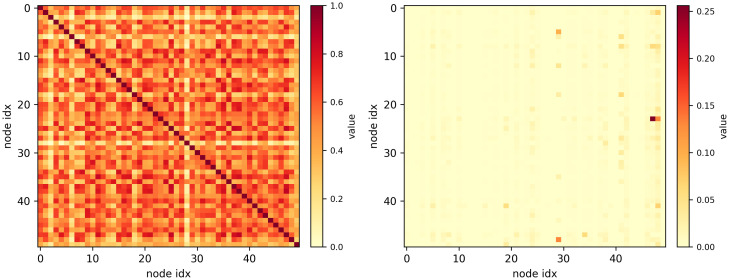
Visualization of ${𝐀}_{\text{MIC}}$ and ${𝐀}_{adp}$.

AMGST aggregates spatial features using both the adaptive matrix and the MIC matrix. To investigate whether these matrices can reflect more realistic spatial correlations, we visualize the two matrices for the top 50 nodes on the PEMS08 dataset, as shown in Fig 6. AMGST is able to uncover long-range spatial correlations: the MIC matrix reveals fine-grained similarity patterns and often assigns high scores to node pairs that are not physically adjacent but exhibit similar traffic dynamics, enabling the model to identify distant nodes with correlated flows. In contrast, the learned adaptive matrix is sparse and concentrates on the most salient connections discovered during training, thereby exposing latent spatial relationships that are not apparent from physical proximity alone; together, these complementary matrices let AMGST capture both detailed functional similarity and concise, interpretable connectivity structure.

## 5. Conclusions

This paper proposes a deep learning model, AMGST, for traffic information prediction. The graph convolution layers employ appropriate matrices to capture both global and local spatial features, while spatial attention is applied after each layer to alleviate the over-smoothing problem. Additionally, temporal attention is used to directly capture the dynamic temporal dependencies. Furthermore, the feature extraction is built upon dynamic inputs, enabling the model to better exploit dynamic spatiotemporal correlations.

The model is comprehensively trained and tested on traffic flow and speed datasets and compared with nine baseline methods. The results demonstrate that, in most cases, AMGST outperforms the majority of existing methods.

Although AMGST emphasizes the dynamic characteristics of traffic data, integrating direct influencing factors such as weather conditions and traffic incidents could further improve prediction accuracy. Moreover, despite its relatively straightforward architecture, the model still entails considerable computational complexity. Future work will focus on developing more efficient methods for dynamic feature extraction.
